# Prognostic value of the C-PLAN index in patients with advanced esophageal cancer treated with immune checkpoint inhibitors

**DOI:** 10.1016/j.clinsp.2026.100917

**Published:** 2026-03-24

**Authors:** Shanshan Shao, Hu Zhang, Yawen Gu, Xiuyuan Yang, Junxing Huang

**Affiliations:** a Department of Oncology, Taizhou People’s Hospital, Taizhou City, Jiangsu Province, China; b Department of Emergency Surgery, Taizhou People’s Hospital, Taizhou City, Jiangsu Province, China

**Keywords:** Esophageal neoplasms, Immunotherapy prognosis, Neutrophil-to-lymphocyte ratio, C-PLAN, Survival analysis

## Abstract

**Background:**

Despite significant advancements in immunotherapy for advanced esophageal cancer, there remains a critical need for reliable biomarkers to predict long-term clinical outcomes. The C-PLAN index, a novel composite score integrating C-Reactive Protein (CRP), lymphocyte dynamics, nutritional risk, and the Neutrophil-to-Lymphocyte Ratio (NLR), was developed to serve as a potential prognostic tool in this setting.

**Methods:**

This study included 228 patients with advanced esophageal cancer who received PD-1/PD-L1 inhibitors between January 2018 and March 2023. Patients were stratified into low-risk (score ≤ 2), Medium-risk (score 3–4), or high-risk (score ≥ 5) groups based on the C-PLAN index. Clinical outcomes, including Overall Survival (OS) and Progression-Free Survival (PFS), were assessed over a 24-month follow-up period. Nonlinear associations between the C-PLAN index and OS/PFS were evaluated using restricted cubic splines. Cox proportional hazards models identified independent prognostic factors for OS, while time-dependent Receiver Operating Characteristic (ROC) curves compared the predictive accuracy of the C-PLAN index with that of NLR.

**Results:**

Of the 228 patients enrolled, 189 were included in the final analysis. Significant differences in baseline characteristics were observed across risk groups: low-risk patients exhibited higher Body Mass Index (BMI) and albumin levels, whereas high-risk patients had larger tumor sizes, elevated CRP, Lactate Dehydrogenase (LDH), NLR, and C-PLAN scores. The median PFS was significantly longer in the low-risk group compared with intermediate- and high-risk groups (*p* < 0.001). A significant nonlinear relationship between the C-PLAN index and both OS and PFS was observed (p for nonlinearity < 0.001). Multivariable analysis identified NLR, poorly differentiated histology, and the C-PLAN index as independent predictors of mortality. Time-dependent ROC analyses revealed that the C-PLAN index demonstrated superior predictive accuracy compared to NLR at both 12-months (Area Under the Curve [AUC], 0.710 vs. 0.496) and 24-months (AUC, 0.897 vs. 0.740).

**Conclusion:**

The C-PLAN index provides a robust and clinically meaningful prognostic tool for patients with advanced esophageal cancer treated with immune checkpoint inhibitors. Its superior predictive performance compared to traditional markers such as NLR highlights its potential utility in risk stratification and guiding treatment decisions.

## Highlights

• C-PLAN integrates CRP, lymphocytes, nutrition, and NLR for prognosis in esophageal cancer.
• Nonlinear link between C-PLAN and survival (*p* < 0.001) shown via spline analysis.
• C-PLAN outperforms NLR in predicting OS/PFS (AUC 0.897 vs. 0.740).
• Independent predictor of mortality, enabling tailored immunotherapy decisions.

## Introduction

Esophageal cancer ranks seventh among the most common malignant tumors globally and is the sixth leading cause of cancer-related mortality.^1^ In China, it remains the fourth leading cause of cancer death, with esophageal squamous cell carcinoma accounting for >90% of all cases.^2^ Although significant advances in diagnostic and therapeutic strategies have improved survival rates, the 5-year overall survival rate for esophageal cancer in China remained around 30% between 2003 and 2015.^3^^,^^4^ Recently, immunotherapy has emerged as a promising new treatment option with substantial clinical efficacy, and it is increasingly regarded as one of the main therapeutic approaches for advanced esophageal cancer.^5^ Immune Checkpoint Inhibitors (ICIs) work by blocking the interaction between Programmed Cell Death-1 (PD-1) or its ligand PD-L1, thereby preventing tumor cells from evading immune surveillance.^6^ Clinical trials have demonstrated that patients with esophageal cancer receiving anti-PD-1/PD-L1 immunotherapy can achieve durable clinical responses and improved outcomes. However, due to inter-patient variability in advanced esophageal cancer, approximately 50% of patients do not benefit from ICI therapy.^7^, ^8^, ^9^ Therefore, identifying reliable biomarkers for monitoring and guiding treatment is crucial.

A recent multi-center retrospective study developed a comprehensive scoring system (named the C-PLAN index), incorporating C-Reactive Protein (CRP), Lactate Dehydrogenase (LDH), albumin, Neutrophil-to-Lymphocyte Ratio (NLR), and performance status, which serves as a potential biomarker reflecting systemic nutritional and inflammatory status. This index was found to predict prognosis in non-small cell lung cancer patients receiving immunotherapy combined with other anti-cancer treatments.^10^ However, its specific association with prognosis in advanced esophageal cancer remains unclear. Therefore, this study aimed to evaluate the prognostic value of the C-PLAN index in patients with advanced esophageal cancer treated with ICIs, with the hope of providing a useful tool for clinical decision-making. The results are presented below.

## Materials and methods

### Study population

This study included patients with advanced esophageal cancer who received Immune Checkpoint Inhibitors (ICIs) at our institution between January 2018 and March 2023. Inclusion criteria were: 1) Diagnosis of advanced esophageal cancer in accordance with the SEOM Clinical Guidelines for Esophageal Cancer (2016),^11^ confirmed by histopathological analysis; 2) Age ≥ 18-years and < 80-years; 3) Primary esophageal tumor without prior treatment with radiotherapy, chemotherapy, immunotherapy, targeted therapy, or anti-angiogenic agents; 4) Expected survival of at least three months. Exclusion criteria included: 1) Ineligibility for ICI due to poor performance status; 2) Presence of immune deficiency or autoimmune disorders; 3) Concurrent organ dysfunction (e.g., heart, liver, lung, or kidney); 4) History of other malignancies; 5) Incomplete clinical data or lack of follow-up. This study adhered to the Strengthening the Reporting of Observational Studies in Epidemiology (STROBE) guidelines for reporting observational research. The study protocol was approved by the institutional ethics committee (202001-14), and written informed consent was obtained from all patients or their legal representatives.

### Treatment regimens

Patients with relatively good performance status received a combination of platinum-based chemotherapy (e.g., paclitaxel plus cisplatin) and ICI therapy with camrelizumab/sintilimab, administered every 21-days as one cycle. Patients with poorer general condition received alternative regimens including single-agent or combination chemotherapies (paclitaxel alone or in combination with platinum/5-fluorouracil-leucovorin-oxaliplatin) plus ICI therapy with camrelizumab/sintilimab, also on a 21-day cycle. In cases of chemotherapy-related toxicities such as pain, vomiting, or severe mucositis, corticosteroids were administered at the discretion of the treating physician. Dexamethasone (5 mg/IV) was given on day one of each chemotherapy cycle.

### Assessment parameters

#### Baseline clinical variables

Clinical data collected included age, gender, Body Mass Index (BMI), smoking history (>5 cigarettes/day for ≥1-year), comorbidities (hypertension, diabetes mellitus, and family history of esophageal cancer), histological type (squamous cell carcinoma, adenocarcinoma, other types), tumor size, location (upper, middle, or lower third of the esophagus), morphology (elevated, ulcerative, or stenotic), TNM stage (III–IV), degree of differentiation, and Eastern Cooperative Oncology Group Performance Status (ECOG-PS).^12^

#### Laboratory assessments

Blood samples were drawn prior to treatment for serum analysis. Neutrophil count, lymphocyte count, hemoglobin levels, and platelet count were quantified using an automated hematology analyzer (Mindray BC5390), and the Neutrophil-to-Lymphocyte Ratio (NLR) was calculated. Serum C-Reactive Protein (CRP), Lactate Dehydrogenase (LDH), Albumin (ALB), Carcinoembryonic Antigen (CEA), Carbohydrate Antigen 19–9 (CA19-9), Squamous Cell Carcinoma antigen (SCC), and Carbohydrate Antigen 50 (CA50) were measured using an automated biochemical analyzer (Mindray BC5390) with radioimmunoassay techniques.

#### C-PLAN index

The C-PLAN index was calculated as a composite score based on five parameters: CRP, LDH, ALB, NLR, and ECOG-PS. Scoring criteria were defined as follows: 1) 0-points for each parameter meeting the reference range (CRP < 1.0 mg/dL or LDH < 223 U/L or ALB ≥ 3.5 g/dL or NLR <3.0 or ECOG-PS ≤ 1); 2) 1-point if any of these parameters exceeded reference thresholds (CRP ≥ 1.0 mg/dL or LDH ≥ 223 U/L or ALB < 3.5 g/dL or NLR ≥ 3.0 or ECOG-PS > 1). Patients were stratified into three groups based on the total score: low-risk group (score ≤ 2), Medium-risk group (scores 3–4), and high-risk group (score ≥ 5).

### Follow-up and disease progression assessment

After ICI treatment, patients were followed up for a period of 24-months using imaging modalities to evaluate disease progression. Response criteria were based on RECIST version 1.1^13^: Complete Response (CR) defined as disappearance of all target lesions with short-axis diameter < 10 mm in any lymph node; Partial Response (PR) defined as ≥ 30% reduction in target lesion size without new lesions; Stable Disease (SD) defined as no change in target lesion size; Progressive Disease (PD) defined as ≥20% increase in target lesion size or the appearance of new lesions. Follow-up data were collected from electronic medical records, with the primary endpoint being Overall Survival (OS), and secondary endpoints including Progression-Free Survival (PFS) and Objective Response Rate (ORR). Patients who did not reach any of the endpoints during follow-up had their final date of observation set as March 31, 2025.

### Statistical analysis

All statistical analyses were performed using *R* version 4.3.0. Categorical variables were presented as n (%) and compared using χ² tests. Continuous variables were summarized as mean ± Standard Deviation (SD) if normally distributed or as median with interquartile range (M, P25–P75) otherwise; group comparisons were conducted using *t*-tests or Mann-Whitney *U* tests accordingly. Kaplan-Meier curves were generated to estimate overall survival, and log-rank tests were used for statistical comparison. Cox proportional hazards models were applied to identify independent prognostic factors. Time-dependent Receiver Operating Characteristic (ROC) curves were constructed to evaluate the predictive performance of the C-PLAN index for OS. A p-value < 0.05 was considered statistically significant.

## Results

**Fig. 1:**
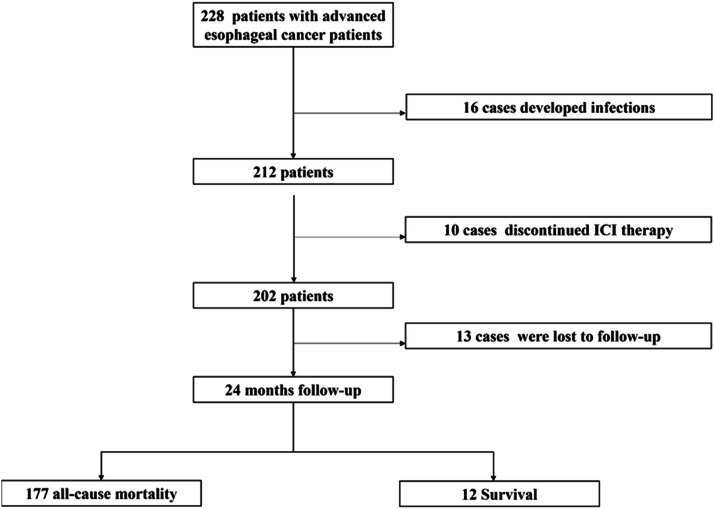
The flowchart of study participants. Note: ICIs, Immune Checkpoint Inhibitors.

Of the 228 patients with advanced esophageal cancer who received Immune Checkpoint Inhibitor (ICI) therapy, 16 developed infections (systemic or localized), 10 discontinued ICI treatment, and 13 were lost to follow-up. After excluding these cases, a total of 189 patients were included in the final analysis (Fig. 1).

The baseline characteristics of the included participants are summarized in Table 1. The low-risk group exhibited significantly higher Body Mass Index (BMI) and Albumin (Alb) levels compared to the medium- and high-risk groups. Conversely, the high-risk group showed significantly elevated tumor diameter, C-Reactive Protein (CRP), Lactate Dehydrogenase (LDH), Neutrophil-to-Lymphocyte Ratio (NLR), and C-PLAN index compared to both the low- and medium-risk groups. No statistically significant differences were observed in other clinical features among the three risk groups.

**Table 1:** Baseline characteristics of included participants.

Clinical features		Overall	Low-risk group	Medium-risk group	High-risk group	p
		*n* = 189	*n* = 49	*n* = 65	*n* = 75	
Age n (%)	< 65 years	82 (43.4)	21 (42.9)	30 (46.2)	31 (41.3)	0.845
	≥ 65 years	107 (56.6)	28 (57.1)	35 (53.8)	44 (58.7)	
BMI (kg/m^2^, median [IQR])		23.10 [21.87, 24.30]	22.23 [21.30, 24.25]	21.87 [20.49, 23.51]	22.55 [21.09, 23.80]	0.022
Gender	Male	91 (48.1)	23 (46.9)	32 (49.2)	36 (48.0)	0.971
	Female	98 (51.9)	26 (53.1)	33 (50.8)	39 (52.0)	
Clinical course (months, median [IQR])		5.00 [5.00, 6.00]	5.00 [5.00, 6.00]	5.00 [5.00, 6.00]	5.00 [4.00, 6.00]	0.645
Tumor diameter (cm, median [IQR])		4.57 [3.78, 5.22]	4.02 [3.31, 4.65]	4.79 [3.96, 5.45]	4.72 [4.13, 5.61]	<0.001
Family history of esophageal cancer (%)	No	152 (80.4)	41 (83.7)	52 (80.0)	59 (78.7)	0.785
	Yes	37 (19.6)	8 (16.3)	13 (20.0)	16 (21.3)	
Smoking history (%)	No	58 (30.7)	11 (22.4)	20 (30.8)	27 (36.0)	0.278
	Yes	131 (69.3)	38 (77.6)	45 (69.2)	48 (64.0)	
Hypertension (%)	No	157 (83.1)	41 (83.7)	55 (84.6)	61 (81.3)	0.868
	Yes	32 (16.9)	8 (16.3)	10 (15.4)	14 (18.7)	
Diabetes mellitus (%)	No	161 (85.2)	42 (85.7)	56 (86.2)	63 (84.0)	0.931
	Yes	28 (14.8)	7 (14.3)	9 (13.8)	12 (16.0)	
Clinical staging (%)	III	73 (38.6)	22 (44.9)	28 (43.1)	23 (30.7)	0.186
	IV	116 (61.4)	27 (55.1)	37 (56.9)	52 (69.3)	
ECOG-PS (%)	No	85 (45.0)	30 (61.2)	30 (46.2)	25 (33.3)	0.009
	Yes	104 (55.0)	19 (38.8)	35 (53.8)	50 (66.7)	
Tumor location (%)	Upper segment	29 (15.3)	10 (20.4)	8 (12.3)	11 (14.7)	0.828
	Middle segment	108 (57.1)	26 (53.1)	39 (60.0)	43 (57.3)	
	Lower segment	52 (27.5)	13 (26.5)	18 (27.7)	21 (28.0)	
CEA (μg/L, median [IQR])		52.56 [46.78, 59.63]	52.15 [46.76, 55.85]	52.02 [45.27, 61.07]	54.61 [48.33, 61.28]	0.098
CA199 (U/mL, median [IQR])		49.28 [42.05, 57.64]	48.18 [42.15, 54.28]	49.28 [41.95, 56.26]	50.01 [42.48, 59.89]	0.321
SCC (ng/mL, median [IQR])		1.25 [1.11, 1.45]	1.19 [1.09, 1.36]	1.25 [1.08, 1.44]	1.32 [1.16, 1.50]	0.068
CA50 (U/mL, median [IQR])		41.87 [36.59, 48.37]	40.58 [34.21, 45.53]	42.66 [38.25, 49.09]	42.58 [37.64, 49.80]	0.17
CRP (mg/dl, median [IQR])		10.77 [9.46, 12.10]	9.07 [7.97, 9.89]	11.17 [10.35, 12.12]	11.55 [10.31, 12.95]	<0.001
LDH (U/L, median [IQR])		223.94 [200.94, 252.83]	201.02 [182.24, 215.22]	221.81 [196.37, 255.85]	248.35 [227.21, 272.13]	<0.001
Alb (g/dL, median [IQR])		3.96 [3.28, 4.58]	4.70 [4.04, 5.07]	4.10 [3.48, 4.55]	3.48 [2.95, 3.92]	<0.001
NLR (median [IQR])		2.47 [2.08, 3.03]	2.03 [1.78, 2.24]	2.58 [2.21, 3.02]	2.82 [2.34, 3.36]	<0.001
Hemoglobin (g/L, median [IQR])		106.70 [96.54, 117.05]	105.87 [96.68, 113.23]	109.11 [96.83, 117.59]	106.19 [96.30, 117.05]	0.691
Platelet count (× 10^9^/L, median [IQR])		272.02 [255.07, 292.31]	280.54 [258.30, 303.95]	271.25 [255.57, 292.31]	270.27 [252.65, 287.66]	0.267
Grade of differentiation	Well-differentiated	125 (66.1)	30 (61.2)	43 (66.2)	52 (69.3)	0.647
	Poorly differentiated	64 (33.9)	19 (38.8)	22 (33.8)	23 (30.7)	
PD-L1 expression (%)	No	52 (27.5)	15 (30.6)	18 (27.7)	19 (25.3)	0.812
	Yes	137 (72.5)	34 (69.4)	47 (72.3)	56 (74.7)	
Malignant tumor type (%)	Ulcerative type	77 (40.7)	19 (38.8)	27 (41.5)	31 (41.3)	0.928
	Raised type	63 (33.3)	16 (32.7)	20 (30.8)	27 (36.0)	
	Infiltrative type	49 (25.9)	14 (28.6)	18 (27.7)	17 (22.7)	
C-PLAN (median [IQR])		4.00 [2.00, 5.00]	2.00 [1.00, 2.00]	4.00 [3.00, 4.00]	6.00 [5.00, 6.00]	<0.001

**Fig. 2:**
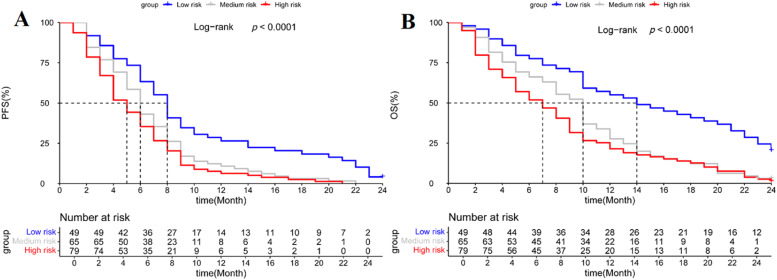
Kaplan-Meier curves of Progression-Free Survival (PFS) and Overall Survival (OS) in advanced esophageal cancer patients receiving immune checkpoint inhibitor therapy, stratified by C-PLAN levels. Notes: Kaplan-Meier curves showing PFS (A) and OS (B) in advanced esophageal cancer patients receiving immune checkpoint inhibitors, stratified by C-PLAN levels.

The Progression-Free Survival (PFS) and Overall Survival (OS) were significantly different among the three risk groups. The low-risk group demonstrated a longer median PFS of 14-months^7^, ^8^, ^9^, ^10^, ^11^, ^12^, ^13^, ^14^, ^15^, ^16^, ^17^, ^18^, ^19^, ^20^, ^21^, ^22^, ^23^, ^24^ and a longer median OS of 8-months,^4^, ^5^, ^6^, ^7^, ^8^, ^9^, ^10^, ^11^, ^12^ both of which were significantly superior to those observed in the medium-risk group (PFS: 10-months^5^, ^6^, ^7^, ^8^, ^9^, ^10^, ^11^, ^12^, ^13^, ^14^, ^15^; OS: 6-months^3^, ^4^, ^5^, ^6^, ^7^, ^8^, ^9^) and the high-risk group (PFS: 7-months^3^, ^4^, ^5^, ^6^, ^7^, ^8^, ^9^, ^10^, ^11^, ^12^, ^13^, ^14^; OS: 8-months.^3^, ^4^, ^5^, ^6^, ^7^, ^8^) Statistical analysis confirmed these differences to be highly significant (log-rank test, *p* < 0.001). The detailed results are presented in Fig. 2.

**Fig. 3:**
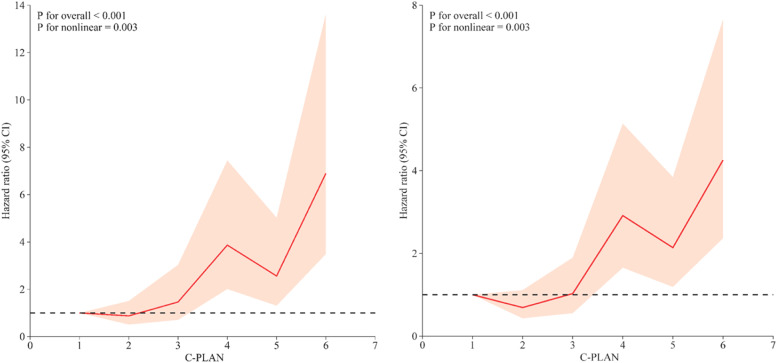
The association of C-PLAN with PFS and OS among Advanced esophageal cancer patients receiving immune checkpoint inhibitor therapy visualized by RCS.

A Restricted Cubic Spline (RCS) analysis was conducted to evaluate the nonlinear relationship between the C-PLAN index and survival outcomes in patients with advanced esophageal cancer treated with Immune Checkpoint Inhibitors (ICIs). The results demonstrated a significant positive nonlinear association of the C-PLAN index with both Progression-Free Survival (PFS) and Overall Survival (OS), with p-values for nonlinearity < 0.001 (Fig. 3A and B).

Multivariable Cox proportional hazards regression analysis was performed to identify independent prognostic factors for Overall Survival (OS) in patients with advanced esophageal cancer treated with ICIs (Table 2). The results of the multivariate analysis demonstrated that three clinical variables were independently associated with worse prognosis: Neutrophil-to-Lymphocyte Ratio (NLR), poorly differentiated histology, and the C-PLAN index. Specifically, elevated NLR was significantly associated with a 39% increased risk of all-cause mortality compared to the reference group (Hazard Ratio [HR = 1.39], 95% Confidence Interval [95% CI: 1.13–1.72], *p* = 0.002). Poorly differentiated histology was associated with a 46% increased risk of all-cause mortality (HR=1.46, 95% CI: 1.07–2.00, *p* = 0.017), while the C-PLAN index showed an even stronger association, with a 46% increased risk of all-cause mortality (HR=1.29, 95% CI: 1.15–1.44, *p* < 0.001).

**Table 2:** Univariate and multivariate Cox regression analysis for overall survival in advanced esophageal cancer patients treated with immune checkpoint inhibitors.

Characteristics	HR (univariable)	HR (multivariable)	HR (final)
CRP	1.14 (1.06‒1.23, *p* < 0.001)	1.03 (0.95‒1.12, *p* = 0.468)	
LDH	1.01 (1.00‒1.01, *p* = 0.001)	1.00 (1.00‒1.01, *p* = 0.307)	
NLR	1.66 (1.36‒2.02, *p* < 0.001)	1.41 (1.14‒1.74, *p* = 0.001)	1.39 (1.13‒1.72, *p* = 0.002)
C-PLAN	1.37 (1.23‒1.52, *p* < 0.001)	1.23 (1.07‒1.41, *p* = 0.004)	1.29 (1.15‒1.44, *p* < 0.001)
Poorly differentiation	1.35 (0.99‒1.85, *p* = 0.056)	1.46 (1.07‒2.00, *p* = 0.018)	1.46 (1.07‒2.00, *p* = 0.017)

**Fig. 4:**
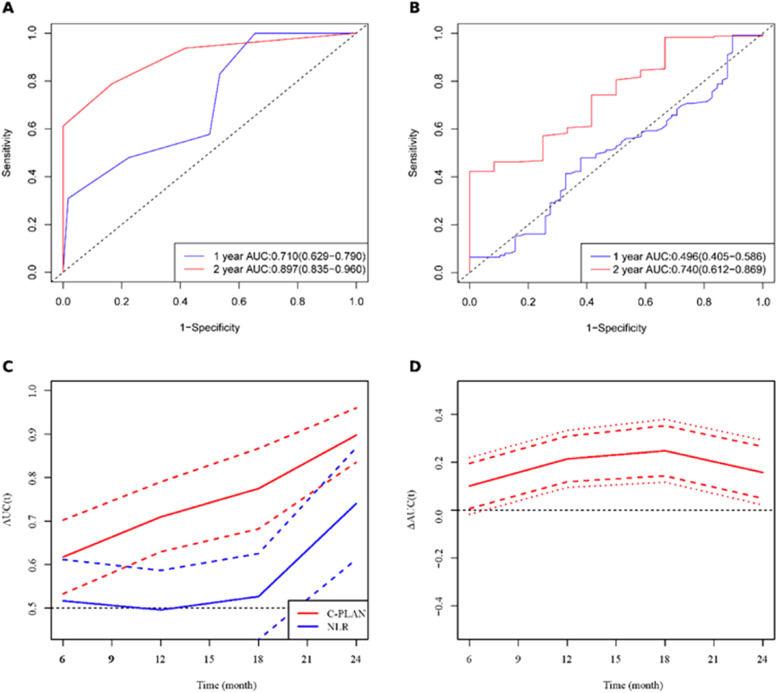
Time-dependent ROC curves and time-dependent AUC for predicting overall survival in advanced esophageal cancer patients receiving immune checkpoint inhibitors therapy using NLR and C-PLAN. Note: Time-dependent ROC curves (A–B), corresponding AUC values (C) for NLR and C-PLAN in predicting overall survival of advanced esophageal cancer patients receiving immune checkpoint inhibitors. (D) Difference in time-dependent AUCs between C-PLAN and NLR over time, illustrating the dynamic discriminatory ability of these markers for predicting outcomes.

A time-dependent Receiver Operating Characteristic (ROC) analysis was conducted to assess the prognostic value of the C-PLAN index compared with the Neutrophil-to-Lymphocyte Ratio (NLR) in predicting Overall Survival (OS) among patients with advanced esophageal cancer receiving Immune Checkpoint Inhibitor (ICI) therapy. The results demonstrated that the C-PLAN index consistently exhibited superior predictive performance over time, with Area Under the Curve (AUC) values of 0.710 and 0.897 at 12- and 24-months, respectively, both significantly higher than those observed for NLR (0.496 and 0.740). Moreover, time-dependent ROC curves showed that the AUC for C-PLAN remained significantly greater than that of NLR across all time points (*p* < 0.05), highlighting its potential as a more robust biomarker for outcome prediction in this patient population (Fig. 4).

## Discussion

Esophageal cancer remains a highly prevalent and aggressive malignancy all over the world, posing significant threats to public health.^14^ The emergence of Immune Checkpoint Inhibitors (ICIs) has introduced new treatment options with the potential to improve survival and quality of life for patients; however, not all patients benefit equally from immunotherapy.^15^^,^^16^ Therefore, developing and validating novel, cost-effective, and non-invasive biomarkers remains a critical priority in clinical practice. While emerging research highlights the potential utility of blood-based inflammatory or nutritional markers as prognostic indicators, their predictive performance has been limited in metastatic Non-Small Cell Lung Cancer (NSCLC) patients receiving first-line ICIs.^17^^,^^18^ A recent multi-center study introduced the C-PLAN index, a novel biomarker integrating Performance Status (PS), inflammation, and nutritional markers, which was found to effectively stratify prognosis in metastatic NSCLC patients undergoing immunotherapy.^19^ However, its prognostic value for advanced esophageal cancer remains controversial, prompting the present investigation into the predictive capacity of the C-PLAN index in esophageal cancer patients receiving immune checkpoint inhibitors.

These findings demonstrated that the C-PLAN index was an independent predictor of Overall Survival (OS), significantly outperforming ECOG performance status and Neutrophil-to-Lymphocyte Ratio (NLR). Notably, the C-PLAN index exhibited a significant nonlinear association with both OS and Progression-Free Survival (PFS) (p for nonlinearity < 0.001), highlighting its potential to capture complex interactions among multiple biomarkers in predicting clinical outcomes. This nonlinear relationship suggests that the C-PLAN index may provide more nuanced risk stratification than traditional linear models, offering a richer understanding of patient heterogeneity.

Furthermore, time-dependent Receiver Operating Characteristic (ROC) analysis revealed that the C-PLAN index demonstrated superior predictive accuracy over NLR at 12- and 24-months, with Area Under the Curve (AUC) values of 0.710 vs. 0.496 and 0.897 vs. 0.740, respectively. These results underscore the temporal utility of the C-PLAN index in assessing prognosis over time, which is critical for guiding treatment decisions and monitoring disease progression in patients receiving ICI therapy. The ability of the C-PLAN index to maintain strong predictive power at both intermediate and long-term follow-up points suggests its potential as a dynamic tool for ongoing clinical evaluation.

The components of the C-PLAN index ‒ C-Reactive Protein (CRP), Lactate Dehydrogenase (LDH), albumin, NLR, and ECOG-PS ‒ have all been previously associated with clinical outcomes in esophageal cancer patients receiving immunotherapy.^20^, ^21^, ^22^, ^23^, ^24^ In advanced cancers, cachexia driven by tumor metabolism and host-tumor interactions significantly impacts patient status and treatment response. Accumulating evidence suggests that systemic inflammation and nutritional status play crucial roles in tumorigenesis; elevated CRP levels and hypoalbuminemia are consistently linked to poor outcomes in cancer patients.^25^^,^^26^ A meta-analysis of 4698 ICI-treated NSCLC patients found that higher pre-treatment CRP was significantly associated with worse OS and PFS,^27^ potentially due to its role in promoting adenosine 2A receptor expression and suppressing T-cell-mediated anti-tumor immunity. Similarly, low albumin levels have been shown to be a key adverse prognostic factor for stage IV NSCLC patients receiving first-line immunotherapy.^28^ Mechanistically, albumin facilitates the catabolism and recycling of IgG antibodies, which are commonly used in ICI therapy, thereby potentially influencing treatment efficacy.^29^

Furthermore, a retrospective study on 252 advanced NSCLC patients treated with pembrolizumab found that NLR was closely associated with survival outcomes,^30^ consistent with the present findings showing a strong association between high NLR and poor OS. Transcriptomic analysis also revealed that elevated NLR correlates with increased expression of immune-related genes such as CD3, SH2D1A, ZAP70, and CD45RA, all involved in immune activation.^31^ In addition, Liu et al. found that neutrophils can secrete vascular endothelial growth factor, promoting tumor angiogenesis and enhancing cell proliferation, migration, and invasion, while lymphopenia weakens T-cell-mediated cytotoxicity against tumor cells.^32^

Similarly, a meta-analysis on the prognostic role of LDH in ICI-treated NSCLC patients showed that elevated pre-treatment LDH levels were significantly associated with poor outcomes.^33^ The present study confirmed this association, possibly due to the acidic microenvironment induced by high lactate production, which promotes tumor invasion and metastasis, thereby negatively affecting patient prognosis.^34^ Moreover, ECOG-PS ≥2 was found to be a predictor of worse outcomes in KRASG12C-mutated NSCLC patients receiving pembrolizumab or combined chemotherapy,^35^ highlighting the importance of PS as a prognostic marker. By integrating and optimizing these predictive factors, the present study demonstrated that the C-PLAN index is an independent predictor of OS in esophageal cancer patients undergoing ICI therapy, offering potential clinical utility for risk stratification.

To further explore the prognostic significance of the C-PLAN index, the authors conducted multivariate Cox regression analysis, and the results showed that the C-PLAN index remained an independent adverse prognostic factor regardless of PD-L1 status or patient age, reinforcing its potential clinical application. Nevertheless, it is important to note that this study was retrospective in nature, and factors such as patient selection, ICI regimens, and treatment strategies may have introduced heterogeneity. Future prospective studies are warranted to validate the dynamic predictive value of the C-PLAN index in monitoring disease progression risk in advanced esophageal cancer patients.

### Limitation

This study has several limitations that should be considered when interpreting the results. First, it is a retrospective analysis conducted at a single institution, which may limit its generalizability to other populations or treatment settings. The relatively small sample size (*n* = 228) could also affect the statistical power to detect subtle prognostic associations, particularly in subgroup analyses. Furthermore, the study did not include patients receiving combination therapies or other systemic treatments, potentially affecting the applicability of the C-PLAN index in more complex clinical scenarios. Additionally, while the index integrates multiple biological and nutritional parameters, it does not account for all potential prognostic factors, such as tumor mutational burden or PD-L1 expression. The follow-up period was limited to 24-months, and longer-term outcomes would be necessary to fully assess its predictive value. Lastly, the study did not explore the underlying mechanisms linking the C-PLAN index to clinical outcomes, which could provide deeper insights into its biological relevance. These limitations highlight the need for further prospective validation in larger, diverse cohorts to confirm the robustness and clinical utility of the C-PLAN index.

In conclusion, the present findings highlight the clinical relevance of the C-PLAN index as a robust predictor of survival outcomes in advanced esophageal cancer patients undergoing ICI therapy. The time-dependent ROC analysis further supports its potential for ongoing risk assessment and treatment decision-making, offering a valuable tool to personalize care and improve patient outcomes.

## Funding

Jiangsu Provincial Medical Innovation Team CXTDA2017042.

## Acknowledgment

Not applicable.

## CRediT authorship contribution statement

**Shanshan Shao:** Writing – review & editing, Writing – original draft, Software, Project administration, Conceptualization. **Hu Zhang:** Writing – review & editing, Writing – original draft, Supervision, Project administration, Methodology, Data curation, Conceptualization. **Yawen Gu:** Writing – review & editing, Writing – original draft, Software, Project administration, Conceptualization. **Xiuyuan Yang:** Writing – review & editing, Supervision, Project administration, Methodology, Formal analysis, Data curation, Conceptualization. **Junxing Huang:** Writing – review & editing, Supervision, Data curation, Conceptualization.

## Conflicts of interest

The authors declare that they have no known competing financial interests or personal relationships that could have appeared to influence the work reported in this paper.

## Data Availability

The datasets used and/or analyzed during the current study are available from the corresponding author on reasonable request.

## Appendix Supplementary materials

Image, application 1
